# Adaptive Ascent Control of a Collaborative Object Transportation System Using Two Quadrotors

**DOI:** 10.3390/s22082923

**Published:** 2022-04-11

**Authors:** Miroslav Pokorný, Jana Nowaková, Tomáš Dočekal

**Affiliations:** 1 Department of Cybernetics and Biomedical Engineering, Faculty of Electrical Engineering and Computer Science, VSB-Technical University of Ostrava, 17. listopadu 2172/15, 708 33 Ostrava-Poruba, Czech Republic; tomas.docekal@vsb.cz; 2 Department of Computer Science, Faculty of Electrical Engineering and Computer Science, VSB-Technical University of Ostrava, 17. listopadu 2172/15, 708 33 Ostrava-Poruba, Czech Republic; jana.nowakova@vsb.cz

**Keywords:** unmanned aerial vehicles, collaborative transportation, admittance force feedback control, adaptation

## Abstract

The paper focuses on the issue of collaborative control of a two quadrotor (Unmanned Aerial Vehicle QDR) system. In particular, two quadrotors perform the task of horizontally transporting a long payload along a predefined trajectory. A leader–follower method is used to synchronize the motion of both QDRs. Conventional PD controllers drive the motion of the leader QDR-L to follow a predefined trajectory. To control a follower QDR-F drive, in the case of indoor applications, a Position Feedback Controller approach (PFC) can be used. To control the QDR-F, the PFC system uses the position information of QDR-L and the required accurate tracking cameras. In our solution, outdoor applications are considered, and usage of the Global Positioning System (GPS) is needed. However, GPS errors can adversely affect the system’s stability. The Force Feedback Controller approach (FFC) is therefore implemented to control the QDR-F motion. The FFC system assumes a rigid gripping of payload by both QDRs. The QDR-F collaborative motion is controlled using the feedback contact forces and torques acting on it due to the motion of the QDR-L. For FFC implementation, the principle of admittance control is used. The admittance controller simulates a virtual “mass-spring-damper” system and drives the motion of the QDR-F according to the contact forces. With the FFC control scheme, the follower QDR-F can be controlled without using the QDR-L positional feedback and the GPS. The contribution to the quality of payload transportation is the novelty of the article. In practice, one of the requirements may be to maintain the horizontal position of the payload. In this paper, an original solution is presented to minimize the horizontal position difference of both QDRs. A new procedure of the transfer admittance controller adaptation according to the mass of the transported payload is designed. The adaptive admittance FFC system is implemented in a Matlab-Simulink environment. The effectiveness of its trajectory tracking and horizontal stabilization functions for variations of the payload mass are demonstrated by numerical calculations.

## 1. Introduction

The development of modern methods and tools of mechatronics is accompanied by a wide development of robot applications in many fields of production and services. Special attention is paid to quadrotors (QDRs), which are used for package delivery [1], wildfire monitoring, and fighting [2], search and rescue operations [3,4,5], disaster management [6] and military activities (MQ1 Predator, Orion, UNITED 40) [7], for example. The use of QDRs in the implementation of such activities brings a significant increase in their efficiency.

However, the use of individual drones is limited by the size and mass of payload transported. Therefore, it is very often necessary to use more cooperating drones to perform tasks in a real environment. The performance of drones can be significantly enhanced by employing collaborative strategies that enable the fulfilment of the transport task using multiple cooperating robots [1,8,9].

Cooperative activities require the solution to several new issues in the fields of automatic control and sensor measurements. Cooperative transport management is addressed, and the control of multiple cooperative quadrotor manipulator systems is discussed by many works [8,9,10]. The motivation of the research work is the development of a reliable and powerful system using multiple mobile robots, which meets the specific requirements of various applications.

Research in the area of collaborative transportation using multiple robots is inspired by approaches such as those used by biological entities (ants) to transport food particles together [11]. The next research has focused on the field of collaborative transport using mobile ground robots [12]. The cooperative lifting of the Ant military robot is described in [13]. To force amplification, a multiple ground-based robotic transport system is presented in [14]. For extending these works, applied to ground as well as aerial robots, it was necessary to address other new issues, such as the highly nonlinear and unstable dynamics of aerial systems and the role of gravity.

The Leader–Follower (LF) control method is an extremely widespread approach to multi-robot collaborative control solutions, based on the principles of swarm robotics [15]. LF control of flight formation of aerial vehicles is presented, for example, in [16]. To apply the LF control method, the position information of the Leader robot is needed. If an indoor application is considered, the motion tracking cameras are used and the Global Positioning System (GPS) in outdoor environments is applied. This is how centralized the Position Feedback Controller (PFC) is defined.

When using the PFC approach, the coordination of movement of the followers is controlled by the position controller. The system uses feedback from the current leader position and generates the desired trajectory for the followers. Their movement is then controlled by linear PID controllers.

The PFC system requires reliable and accurate robot position information obtained from the cameras to monitor their movement (tens of millimeters). The motion tracking cameras meet this requirement, and the PFC approach is therefore especially suitable for indoor applications.

Outdoor applications use the Global Positioning System (GPS) to obtain location information. Commercial GPS systems measure the position of an object with an error of 1 to 2 m, and the differential GPS system works with an accuracy of the order of centimeters. Such errors in the PFC management system would cause instability due to the large interaction forces that would arise in the collaborative transport of the payload. Therefore, the accuracy of the GPS system does not guarantee either the observance of the prescribed trajectory or the stability of the collaborative system.

The use of the Force Feedback Controller (FFC) control approach is a solution to this problem. When using the FFC approach, the synchronization of drones in trajectory tracking and the stability of the system of drones during the performance of the transport task is ensured based on the information on physical interactions between the individual drones and the payload transferred.

The coordination of the movement of the followers is controlled by feedback control, which uses information about the interaction forces and torques acting, through the payload, on the followers due to the movement of the leader. The solution does not require position information to control the movement of the followers, but it assumes a fixed connection between the drones and the payload and the placement of force measurement sensors on the drone effector, i.e., at the contact points of the QDRs and the payload. The FFC system therefore assumes a fixed grasping between the robots and the payload. The FFC approach is also suitable for outdoor applications [1,9].

The FFC approach is based on the principle of minimization of contact force and torques, respectively. The FFC control is addressed in [17]. A collaborative transportation system using micro-aerial vehicles within the Kalman filter is used to estimate the contact forces. The solution employs an inertial visual navigation system and can be implemented only in outdoor applications.

The principle of passive control in collaborative payload transport is discussed in [18]. The implementation of FFC using fuzzy logic-based controllers is discussed in [19,20]. The solution requires the use of a visual inertial navigation system and is therefore limited to the indoor environment.

Article [1] discusses the issue of payload transport and its grasping by both QDRs. The article presents a dynamic model of a pair of QDRs in the case of a firm grip. The work uses a combination of centralized and decentralized management. The mass of the QDRs is assumed to be greater than the weight of the payload.

The solution in work [9] assumes collaborative transportation of a payload suspended by cables. An analytical solution is presented for tension constraints and stability. To avoid the collisions, several assumptions are defined regarding cable tension and geometry of system configuration.

The issue of cooperative transportation of a cable-suspended payload by multiple quadrotors is also addressed in [21]. The differential flatness concepts for both point mass and rigid body payloads are discussed. The cable tension issue is addressed. A case, where the tensions of cables are non-negative including the case with zero tensions, is considered.

The inverse kinematics issue of a collaborative QDRs transport within a cable suspended payload is dealt with in [22]. The position of QDRs is determined where the payload position and orientation are known.

In all these solutions using the cable-suspended payload, cable tension acts as a constraint and must be a known parameter to control the system. Therefore, instability of the system can occur, when incorrect tensions are assumed in calculations. An approach, using transportation of a payload suspended by cables, is presented in [9,21]. This solution requires fulfilment of conditions of cable tension and geometry. The solution in [21] introduces the FFC control approach, where Kalman filtering is used to estimate the contact forces acting on robots manipulating a workpiece collaboratively.

In Ref. [23], a linear PID controller is used to control each individual quadrotor and a slide mode controller is used to address the issue of flying in the formation, which uses the leader–follower structure for three quadrotors. The leader flies along a predefined trajectory, which is determined by the trajectory tracking controller. The formation controller is designed to maintain a certain shape on the *xy* plane, according to the relative kinematics between the leader and the follower. After the formation controller generates the speed for the follower, the follower can track this speed so as to keep the relative distance and the orientation angle constant at the same height. The leader follows the predefined formation trajectory, while the follower tracks the desired speed to maintain the shape.

The basic issue of force and moments control for multiple cooperative robots manipulating a workpiece collaboratively is addressed in [19]. The solution uses the leader–follower approach. External acting forces are estimated using force and torque sensors. The control scheme uses fuzzy controllers. The Kalman filter is used to estimate the force acting on the effector. The parameters of fuzzy controllers applied in this paper are identified using a neural network in [20]. The principle of force and torque minimization to achieve collaborative motion is applied.

FFC control addresses the issue of using a collaborative transport system of QDRs in an outdoor environment with regard to eliminating the need for accurate measurement of the current position of QDR-F. Further progress in the application of the collaborative FFC management system in the real outdoor environment is covered in [18], which proposes the use of admittance FFC management approaches.

The admittance control strategy considers force as an input and provides motion as an output, whereas the impedance base control strategy takes motion as an input and provides force as an output. The principles of impedance and admittance controllers are proposed for the control of robots interacting with the environment. Hybrid control of impedance–admittance control of robot manipulators in the mode of interaction with the environment is discussed in [24].

The admittance controller is a type of compliance control, where the force is the input, and the output is the corresponding trajectory [25]. The movement of the QDR-F is controlled by the interaction forces and moments that act on it due to the movement of the QDR-L is the output. The movement of QDR-L is controlled along a predefined trajectory by PID controllers. The QDR-F control strategy is to minimize the forces acting on it to achieve synchronous motion with the QDR-L.

The admittance controller drives the movement of the QDR-F in each coordinate axis. The required trajectory QDR-F is generated in accordance with the acting contact forces through a virtual system spring-mass-damper (second order admittance control law). The admittance controller then provides the follower with the desired behavior in terms of the required force compliance and generates the required trajectory for QDR-F. Ref. [26] is focused on controlling a manipulator attached to a quadrotor using the admittance control approach. The paper presents the design and control of a multirotor-based aerial manipulator developed for outdoor operation. The multi-rotor has eight rotors and a large payload to integrate a 7-degrees of a freedom arm and to carry sensors and processing hardware needed for outdoor positioning. The paper focuses on the control design and implementation aspects. A stable backstepping-based controller for the multirotor that uses the coupled full dynamic model is proposed, and an admittance controller for the manipulator arm is outlined. Due to the admittance controller, the dynamics of the system changes to maintain its stability during the arm movement.

Ref. [10] is focused on a multi-quadrotor manipulator system. The three-layers hierarchical control is developed to endow the common grasped object with a user-specified desired behavior (trajectory tracking, compliant interaction). The control system is based on the admittance control approach.

The admittance control system is presented in application of physical-human quadrotor interaction in [27]. The admittance controller includes the virtual “mass-spring-damper” system. The admittance controller generates a reference motion of the quadrotor, which is tracked by a PID controller. A similar solution is used to implement admittance control in the presented contribution.

Collaborative transportation using a multi-quadrotor is presented in [17]. The solution uses a leader–follower approach, and the admittance controller is applied to control the follower. The Kalman filtering is used to estimate the contact forces. Nevertheless, the control scheme relies on visual inertial navigation, and, in outdoor applications, the GPS is needed.

In [18], the very effective admittance-based force control for collaborative transportation using two QDRs is discussed. The leader–follower principle is used to design the two-QDRs system using admittance control of the follower. As a benefit, this system acts without relying on the GPS and allows the implementation in the real outdoor world. Such an approach is used as the basis for the solution in our paper. The solution proposed in [18] does not depend on load geometry, cable tension, constraints, or the grasping point position. If no remote control is used, the GPS can be used to measure position.

The time of the control process, which is required as a minimum, is a standard test criterion for assessing the quality of payload transporting along a prescribed trajectory.

In some real-world tasks, such as search and rescue operations, disaster management, and special package delivery, the transport system must meet additional specific requirements. For example, transport of injured persons or liquids requires movement without sudden changes in the speed or direction, and transport in a tighter horizontal position.

The smooth shape of the motion trajectory in the presented paper is addressed by adjusting the step change of the QDR position by a second-order shaping circuit within adjustable parameters. This solution ensures smooth transport of the load in the horizontal plane, without sharp or large inclinations of the payload in the *x-* and *y*-axes. The presented paper addresses the issue of horizontal stability of the payload depending on its mass. The degree of the horizontal position of the payload is measured by the difference (error) of the height coordinates of the gripping points of its ends (payload tilt error). The error minimization requirement determines the requirements for the coordination of the movement of both QDRs when performing the movement along the required vertical trajectory, namely in the phase of their ascent.

To minimize the error of the horizontal position of the payload, the optimization problem has been formulated and the original procedure of adapting the transmission of the admittance ascent regulator QDR-F depending on the current weight of the transported payload has been designed.

The effectiveness of the proposed procedures was verified using a QDR-L/QDR-F system model and numerical simulations.

## 2. Issue Formulation

**Figure 1:**
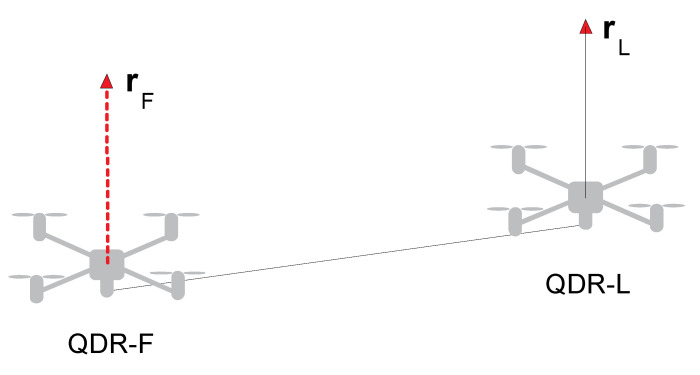
Collaborative transportation system configuration [18].

The topic is defined by addressing the issue of ascent and transporting a beam shaped payload of mass mP using two QDRs [18]. The leader–follower approach is used to address the collaborative payload transport issue. One QDR-L acts as the Leader (QDR-L), and the other acts as the Follower (QDR-F). The solution aims at steering the QDR-L along the desired 3D trajectory. The configuration of the system is shown in Figure 1.

The Follower tracks the Leader’s trajectory based on the contact forces acting on it due to the Leader’s motion. The system has rigid connections—both QDRs are rigidly attached to the payload. Despite the rigid QDRs’ attachment to the payload, the QDR-L exerts a contact force on the QDR-F, which is an input to its attitude controllers. The control strategy of the QDR-F is to compensate the contact forces, thus achieving collaborative control, i.e., synchronization of the motion of both QDRs.

The QDR-L is equipped with feedback controllers for its attitude. The attitude control is performed by changing the roll, pitch, and yaw angles along the x-,y-, and z-axes and the acceleration along the z-axis [8]. It is assumed that the yaw rotation of both QDRs around the z-axis is constant ($\psi_{L}$ = $\psi_{F}$ = 0) in our solution.

The role of collaborative control is extended by stabilizing the beam-shaped payload’s horizontal position during changes in the mass of the payload by minimizing its roll. It is achieved by adapting the proportional gain of the admittance controller driving the motion of the QDR-F in the z-axis direction.

The control system of both QDRs is equipped with PD controllers. The real rigid QDR-L-PAYLOAD-QDR-F system is equipped with contact force sensors on the gripping side of the QDRs (Section 7). A dynamic mathematical model calculates the contact forces in the computer simulation system. The Takagi–Sugeno fuzzy model approximates the nonlinear adaptation function [28].

## 3. QDRs Control System Design

The collaborative control strategy for transport tandem QDRs uses a distributed leader–follower control approach. The synchronization of QDR-L and QDR-F in tracking the desired motion trajectory and the stability of the QDRs system during the transport task is ensured in the FFC (Force Feedback Control) system based on the information about the physical interactions between the individual QDRs and the payload transported. In order to achieve synchronization of their motion, the QDR-F must compensate (minimize) the interactive contact force with its motion and thus maintain an equilibrium position.

**Figure 2:**
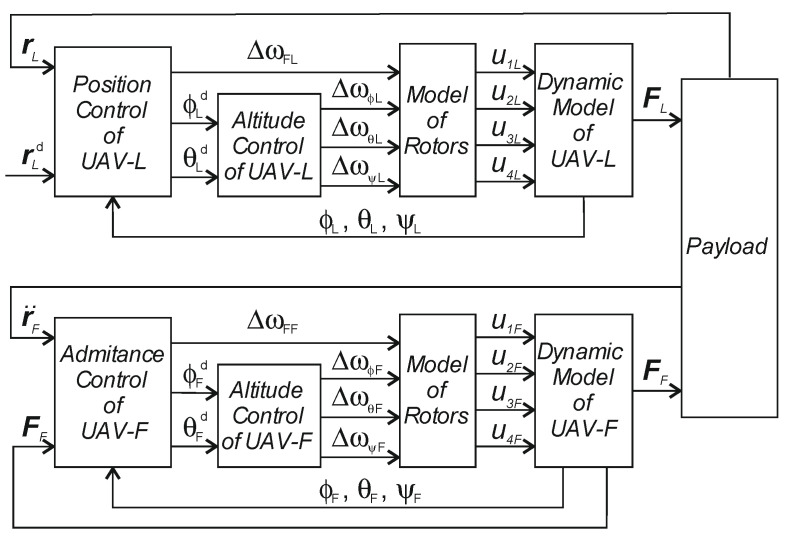
Block diagram of the collaborative QDR-L/QDR-F system.

The principal block diagram of the leader–follower collaborative transport control system is shown in Figure 2. The control scheme of both QDRs contains an outer control loop and an inner control one. The outer control loop of the QDR-L includes PD controllers for the motion of the QDR-L along a specified trajectory $r_{L}^{d}$, whose action variables $\phi_{L}^{d}$, $\theta_{L}^{d}$ are input as rotation setpoints about the x- and *y*-axes to the PD controllers of the inner loop. The action magnitude $\Delta \omega_{FL}$ of the linear motion controller along the z-axis enters directly into the rotors model block of QDR-L. Design and Lyapunov stability analysis is presented in [29]. A non-conventional variable is introduced to study the stability analysis of QDR when controlled by a PID position controller.

The inner loop computational block Model of Rotors calculates, from the action magnitudes of the controllers, the magnitudes of the forces and torques acting in each axis. The forces and torques are the QDR-L dynamic model inputs. QDR-L outputs represent information about the position of the QDR-L in each axis and the magnitude of the contact forces $F_{L}$. These contact forces are measured by sensors at the rigid payload grip locations in the real system (see Section 7).

The QDR-L contact forces and acceleration values acting on the payload and QDR-F are input to the admittance controllers of the QDR-F outer control loop (Figure 2). The admittance controllers generate the QDR-F reference trajectory using the action variables $\phi_{F}^{d}$, $\theta_{F}^{d}$, and $\Delta \omega_{FF}$. These action variables enter to the model of the rotors of QDR-F as in the case of the QDR-L model.

**Figure 3:**
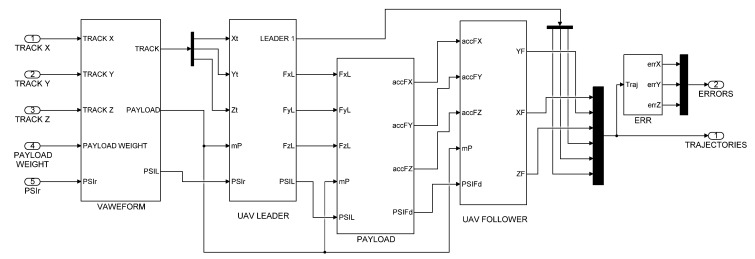
Simulation scheme of the QDR-L/QDR-F system.

The simulation scheme of the collaborative transport QDR-L/QDR-F system is shown in Figure 3. In contrast to the block diagram in Figure 2, the step changes of the setpoints are adjusted here by second-order transmission circuits. This modification ensures that the desired trajectory of the QDR-L is tracked without abrupt changes in its speed and direction. This requirement is, due to the motion strategy of the rigid QDRs system, in the mode of stabilizing the horizontal position of the payload by minimizing the difference (error) of its inclination.

### 3.1. Leader QDR-L Control

The leader QDR-L moves along a prescribed trajectory $r_{L}^{d}$ (1)—Figure 2. The motion control is made by changing the roll angles about the x (roll—φ), y (pitch—θ) axes and acceleration in the z-axis direction ${\ddot{z}}_{zL}^{d}$. The rotation of both QDRs about the z-axis (yaw—ψ) is zero. The control objective of QDR-F is to minimize the contact forces acting at the point of its rigid connection with the payload.

The control of QDR-L is the source of the collaborative motion of the tandem. The components of the desired trajectory vector of QDR-L in the axes are

(1) $$r_{L}^{d}={\left[\begin{array}{ccc} x_{L}^{d} & y_{L}^{d} & z_{L}^{d} \end{array}\right]}^{T}.$$

A schematic of the inner and outer control loops is given in Figure 2. The controllers for the position of QDR-L in the x- and y-axes in the outer control loop use PD controllers

(2) $${\ddot{r}}_{L}^{d}=k_{P}*\left(r_{L}^{d}-r_{L}\right)+k_{D}*\left({\dot{r}}_{L}^{d}-{\dot{r}}_{L}\right).$$

The $k_{P}$ and $k_{D}$ gains are tuned expertly. The required roll and pitch angles to control the QDR-L position on the  x and y axes are calculated using the linearized dynamic equations [18]:

(3) $$\phi_{L}^{d}=\frac{1}{g}\left({\ddot{x}}_{L}^{d}sin\psi_{T}-{\ddot{y}}_{L}^{d}cos\psi_{T}\right),\;\theta_{L}^{d}=\frac{1}{g}\left({\ddot{x}}_{L}^{d}cos\psi_{T}+{\ddot{y}}_{L}^{d}sin\psi_{T}\right).$$

The change in rotor rotation speed for controlling the position of QDR-L on the z-axis is calculated by the relation

(4) $$\Delta \omega_{FL}=\frac{m_{L}{\ddot{z}}_{L}^{d}}{8k_{F}\omega_{hL}}.$$

The nominal rotational speed of the rotors to reach the hover state of the QDR in the case of equal mass *m* of both QDR-L and QDR-F is given by

(5) $$\omega_{hL}=\sqrt{\frac{mg}{4k_{F}}}.$$

PD controllers of the inner control loop are used to guide the motion of QDR-L in the X, Y axes and rotation on the *z*-axis

(6) $$\Delta \omega_{\phi L}=k_{P\phi }\left(\phi_{L}^{d}-\phi_{L}\right)+k_{D\phi }\left({\dot{\phi }}_{L}^{d}-{\dot{\phi }}_{L}\right),\;\Delta \omega_{\theta L}=k_{P\theta }\left(\theta_{L}^{d}-\theta_{L}\right)+k_{D\theta }\left({\dot{\theta }}_{L}^{d}-{\dot{\theta }}_{L}\right),\;\Delta \omega_{\psi L}=k_{P\psi }\left(\psi_{L}^{d}-\psi_{L}\right)+k_{D\psi }\left({\dot{\psi }}_{L}^{d}-{\dot{\psi }}_{L}\right).$$

The required values of the rotational speed of the four rotors of QDR_L

(7) $$\omega_{1L}=\omega_{hL}+\Delta \omega_{FL}-{\Delta \omega }_{\theta L}+{\Delta \omega }_{\psi L},\;\omega_{2L}=\omega_{hL}+\Delta \omega_{FL}+{\Delta \omega }_{\phi L}-{\Delta \omega }_{\psi L},\;\omega_{3L}=\omega_{hL}+\Delta \omega_{FL}+{\Delta \omega }_{\theta L}+{\Delta \omega }_{\psi L},\;\omega_{4L}=\omega_{hL}+\Delta \omega_{FL}-{\Delta \omega }_{\phi L}+{\Delta \omega }_{\theta L}-{\Delta \omega }_{\psi L}.$$

The traction forces and torques of four identical QDR-L rotors are calculated in the block Model of Rotors using their characteristics, i=1,…,4

(8) $$F_{iL}=k_{F}\omega_{iL}^{2},\;M_{iL}=k_{M}\omega_{iL}^{2}.$$

The forces and torques acting on the QDR-L are calculated using the following relations

(9) $$u_{1L}=-F_{2L}\cdot l+F_{4L}\cdot l,\;u_{2L}=F_{1L}\cdot l-F_{3L}\cdot l,\;u_{3L}=-M_{1L}+M_{2L}-M_{3L}+M_{4L},\;u_{4L}=F_{1L}+F_{2L}+F_{3L}+F_{4L}.$$

The quantities $u_{1L}$—the torque acting on the QDR-L on the x-axis; $u_{2L}$—the torque acting on the QDR-L on the y-axis; $u_{3L}$—the torque acting on the QDR-L on the z-axis; $u_{4L}$—the total tractive force acting on the QDR-L on the z-axis. $u_{1L}$, $u_{2L}$, $u_{3L}$, and $u_{4L}$ are the input variables of the dynamic mathematical model of the QDR-L.

### 3.2. Follower QDR-F Control

**Figure 4:**
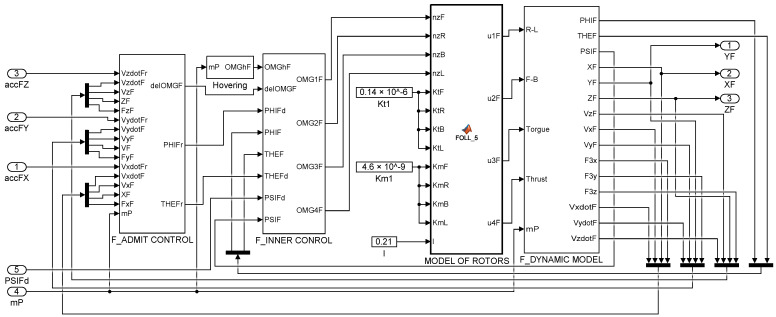
Simulation scheme of QDR-F.

The controllers and computational blocks of the inner control loop of QDR_F (Equations (3)–(9)) are identical to those of QDR_L. The schematic of the QDR-F simulation model is extended with an admittance controller block to represent the outer control loop compared to the QDR-L model (Figure 4).

**Figure 5:**
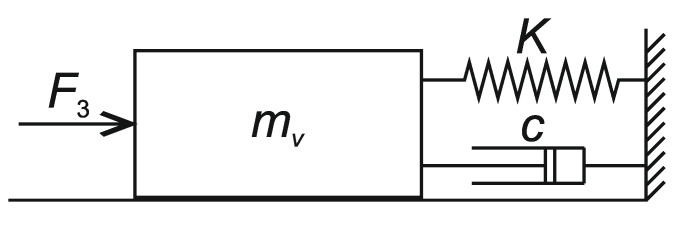
Virtual dynamic system.

For the synchronization improvement of the motion of QDR-L and QDR-F, the FFC system is extended to AFCC (Admittance Force Feedback Control). In the admittance system, the desired motion for QDR-F is further corrected based on the interaction contact forces generated by their transmission to QDR-F due to the motion of QDR-L. A “mass-spring–damper” simulation circuit adjusts the dynamics of the compensation process with a second-order inertial transfer (Figure 5) for the trajectory tracking accuracy and system stability. The calculation of the required compensation force is performed separately in each coordinate axis of the QDR-F. The compensation force waveform shall be adjusted to maintain the simulation system in an equilibrium position in which the springs K are not stretched and the dampers c are not activated [18].

The admittance controllers are implemented in all axes in the QDR-F motion control system. The law of admittance control is given by [18]

(10) $$m_{v}\left({\ddot{r}}_{d}-{\ddot{r}}_{f}\right)+c\left({\dot{r}}_{d}-{\dot{r}}_{f}\right)+K\left(r_{d}-r_{f}\right)=F_{F}$$

where ${\ddot{r}}_{f}$, ${\dot{r}}_{f},$ and $r_{f}$ represent the modified (reference) trajectory of the QDR-F motion, its first and second derivatives, and the force $F_{F}$ is the contact force calculated by the dynamic model. The transfer parameters are: K—spring stiffness, c—the damping coefficient and $m_{v}$—a virtual mass of the admittance system. The parameters of the shaping circuit are tuned separately for each axis.

The admittance PD controllers control the motion of the QDR-F along the reference trajectory by their outputs $\phi_{F}^{d}$, $\theta_{F}^{d}$, and $\psi_{F}^{d}$, which are the inputs to the internal control loop of the QDR-F. The parameters of the admittance controllers for driving the motion along the x- and y-axes are tuned expertly. The proportional gain of the admittance controller for driving the motion along the z-axis is automatically adapted to the actual mass of the payload (Section 5).

The proportional gain of the z-axis attitude controller is adapted (concerning the requirement of minimizing the QDR-F and QDR-L attitude error for mP load mass changes) by an adaptive fuzzy TS model (21). The output of the dynamic admittance control terms of QDR-F

(11) $$ADMx=m_{Vx}\left({\ddot{x}}^{f}-{\ddot{x}}_{F}\right)+c_{x}\left({\dot{x}}^{f}-{\dot{x}}_{F}\right)+K_{x}\left(x^{f}-x_{F}\right),\;ADMy=m_{Vy}\left({\ddot{y}}^{f}-{\ddot{y}}_{F}\right)+c_{y}\left({\dot{y}}^{f}-{\dot{y}}_{F}\right)+K_{y}\left(y^{f}-y_{F}\right),\;ADMz=m_{Vz}\left({\ddot{z}}^{f}-{\ddot{z}}_{F}\right)+c_{z}\left({\dot{z}}^{f}-{\dot{z}}_{F}\right)+K_{z}\left(z^{f}-z_{F}\right).$$

Admittance PD controllers generate the setpoints for the QDR-F inner loop controllers

(12) $$\phi_{F}^{d}=Px\left(ADMx-F_{F}x\right)+Dx\left(\dot{ADMx}-\dot{F_{F}x}\right),\;\theta_{F}^{d}=Py\left(ADMy-F_{F}y\right)+Dy\left(\dot{ADMy}-\dot{F_{F}y}\right),\;\Delta \omega_{FF}=Pz\left(ADMz-F_{F}z\right)+Dz\left(\dot{ADMz}-\dot{F_{F}z}\right).$$

PD controllers of the internal control loop are used to control the movement of QDR-F on the x- and y-axes and rotation on the z-axis:

(13) $$\Delta \omega_{\phi F}=k_{P\phi }\left(\phi_{F}^{d}-\phi_{F}\right)+k_{D\phi }\left({\dot{\phi }}_{F}^{d}-{\dot{\phi }}_{F}\right),\;\Delta \omega_{\theta F}=k_{P\theta }\left(\theta_{F}^{d}-\theta_{F}\right)+k_{D\theta }\left({\dot{\theta }}_{F}^{d}-{\dot{\theta }}_{F}\right),\;\Delta \omega_{\psi F}=k_{P\psi }\left(\psi_{F}^{d}-\psi_{F}\right)+k_{D\psi }\left({\dot{\psi }}_{F}^{d}-{\dot{\psi }}_{F}\right).$$

QDR-F rotor speed setpoints, QDR-F rotor traction forces and torques, and QDR-F forces and torques are calculated according to Equations (7)–(9).

## 4. QDR Dynamics Model Design

**Figure 6:**
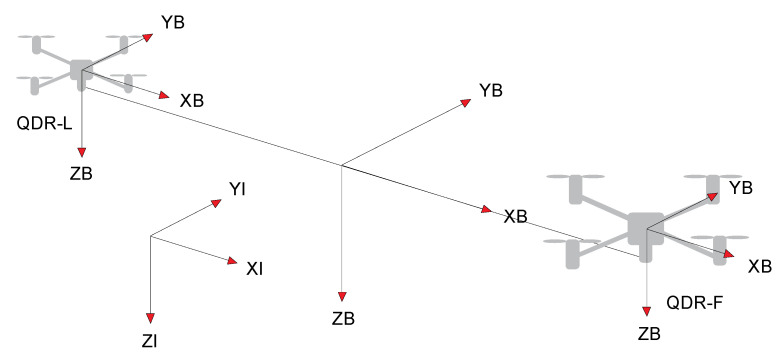
Reference frames [30].

To describe the system’s motion in the free environment, two reference coordinate systems (frames) are considered—see Figure 6 [8]. The coordinate system gives the Earth (inertial) frame with *XI*, *YI* and *ZI* axes. This frame is tightly coupled to the Earth coordinate system. The body frame of QDRs is oriented in a coordinate system, with *XB*, *YB,* and *ZB* axes (body frame). This frame is tightly coupled to the orientation of the arms of the QDRs. The QDR-L, QDR-F and payload body frames have the same orientation due to the rigid coupling of the transport system. The rotational and translational accelerations are modelled within the QDRs bodies (body frame), and the tilts of the QDRs axes and the gravitational force are modelled within the Earth (inertial frame).

**Figure 7:**
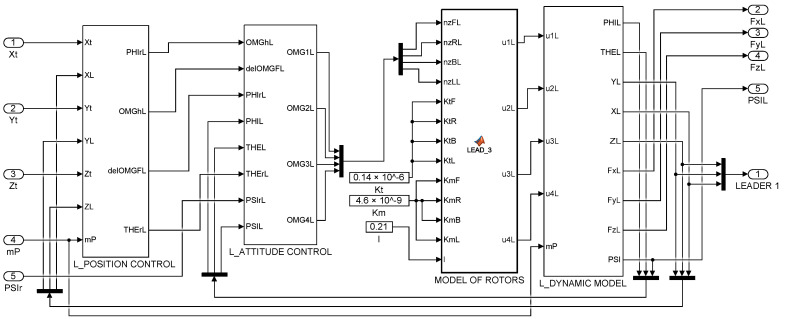
Simulation scheme of QDR-L.

The QDRs Dynamic Model (Figure 4 and Figure 7) is intended to provide a simulation framework that allows the calculation of accelerations, contact forces and torques based on the action quantities of the QDR motion controllers along the prescribed trajectories. The inputs to the dynamic model of QDRs are the forces and torques applied by the propeller to the QDR ($u_{1}$ is the X-axis torque, $u_{2}$ is the Y-axis torque, $u_{3}$ is the Z-axis torque, and $u_{4}$ is the z-axis thrust force) [31]. The rotational and translational accelerations for each axis are transformed from the QDR body frame to the Earth inertial frame by the transformation matrix R during the calculation [17]. The output of QDR-L represents the contact forces acting on QDR-F. In a real system, these forces are measured by suitable sensors at the point of attachment of the payload (Section 7).

The QDR simulation dynamic mathematical model calculates the values of the variables used as their actual values in the inner and outer control loop controllers and monitoring its motion. The angular accelerations of the QDR rotation about the x, y- and z-axes are given by the relations:

(14) $${\dot{\omega }}_{x}=\frac{u_{1}+\left(I_{yy}-I_{zz}\right)\omega_{y}\omega_{z}}{I_{xx}},\;{\dot{\omega }}_{y}=\frac{u_{2}+\left(I_{zz}-I_{xx}\right)\omega_{x}\omega_{z}}{I_{yy}},\;{\dot{\omega }}_{z}=\frac{u_{3}+\left(I_{xx}-I_{yy}\right)\omega_{x}\omega_{y}}{I_{zz}}.$$

By integrating the values of the angular accelerations ${\dot{\omega }}_{x}$, ${\dot{\omega }}_{y}$ and ${\dot{\omega }}_{z}$, we obtain the angular velocities $\omega_{x}$, $\omega_{y}$ and $\omega_{z}$. By transforming the angular velocities from the body frame to the inertial frame and further integration, we obtain the actual values of the angular rotations φ and θ and ψ about the x-, y- and z-axes for the inner loop controllers.

The acceleration of translational motion on the x-,y, and z-axes is given by

(15) $${\dot{v}}_{x}=\omega_{y}v_{z}+\omega_{z}v_{y}-g\sin\theta ,\;{\dot{v}}_{y}=\omega_{x}v_{z}+\omega_{z}v_{x}+gc\cos\theta \sin\emptyset ,\;{\dot{v}}_{z}=\frac{m\omega_{x}v_{y}+m\omega_{y}v_{z}+mg\cos\theta \cos\emptyset -u_{4}}{m}.$$

Transforming the translational velocities from the body frame to the inertial frame and further integrating, we obtain the actual x, y and z values of the QDR positions on the x, y− and z-axes. By integrating the translational accelerations ${\dot{v}}_{x}$, ${\dot{v}}_{y}$ and ${\dot{v}}_{z}$, we obtain the translational velocities $v_{x}$, $v_{y}$ and $v_{z}$. The translational acceleration values of QDR-F ${\ddot{r}}_{f}$ and the force $F_{F}$ enter the admittance controllers of QDR-F.

The contact forces exerted by the QDR-L motion at the point of its grasping are calculated by the dynamic model using equations:

(16) $$F_{xL}=u_{4L}\sin\theta \cos\emptyset ,\;F_{yL}=-u_{4L}\sin\theta ,\;F_{zL}=\cos\theta \cos\emptyset u_{4L}-\left(mQ+mP/2\right)g.$$

In the real system, these forces are measured by sensors (Section 7). As both QDRs are rigidly attached to the payload, the same contact force $F_{F}$ acts on the QDR-F. To achieve the collaborative motion both of QDRs, the QDR-F compensates for this force. The contact acceleration acting on the QDR-F and measured at the point on its grasping is calculated in the simulation mode by the equation

(17) $${\ddot{r}}_{F}=F_{F}\left(mQ+mP/2\right),$$

where mQ and mP are the mass of QDR and the mass of the payload, respectively. Contact forces $F_{F}$ and acceleration ${\ddot{r}}_{F}$ are used as inputs to the admittance controller of QDR-F.

## 5. Payload Horizontal Position Maintenance

The presented paper addresses the issue of maintaining the horizontal position of the payload, namely when the ascent and horizontal movement is performed. The measure of the horizontal position of the payload is defined by the difference in height of the leader and follower grasping points, calculated as an attitude error

(18) $$Err_{z}=z_{L}-z_{F}.$$

To maintain the horizontal position of the payload, an optimization problem has been formulated: minimize the function of error $Err_{Z}$ and the size of proportional gain of the admittance controller $P_{Z}$ by systematically choosing the $P_{Z}$ from the set of its permitted values, and computing the value of the function

(19) $$Err_{Z}=f\left(P_{Z}\right)\;Err_{Z}\to min.$$

Further calculations have confirmed that Function (19) is nonlinear with an extreme of minimum. Equation (19) is an objective function of the optimization problem. 

**Figure 8:**
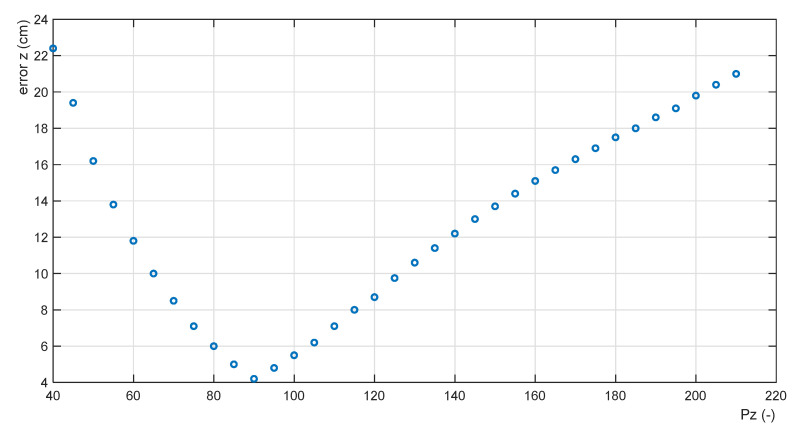
Objective function for mP = 5 kg.

The objective Function (19) must be calculated for each payload mass *mP* from the set of its permitted values. To obtain the objective Function (19), the set of feasible solutions of the optimization problem is reduced to a discrete one. Function (19) has been calculated for the gain range $P_{Z}\in \langle 40;210\rangle$, step $\Delta P_{Z}=5$. The calculations for the value of *mP* = 5 kg is given in Table 1; the shape of the objective function is shown in Figure 8.

The presented function exhibits a minimum $Err_{Z},min$ = 4.2 cm for an optimal proportional gain value $P_{Z},opt$ = 90. Altogether, 21 objective functions have been calculated for mP∈〈0kg; 5kg〉 (the step values of 0.25 kg). This earned 21 points of the discrete function

(20) $$P_{Z},opt=f\left(mP\right)$$

which is a function for the adaptation of the proportional gain $P_{Z}$ of the ADM admittance controller. The data of the discrete Function (20) are listed in Table 2.

**Figure 9:**
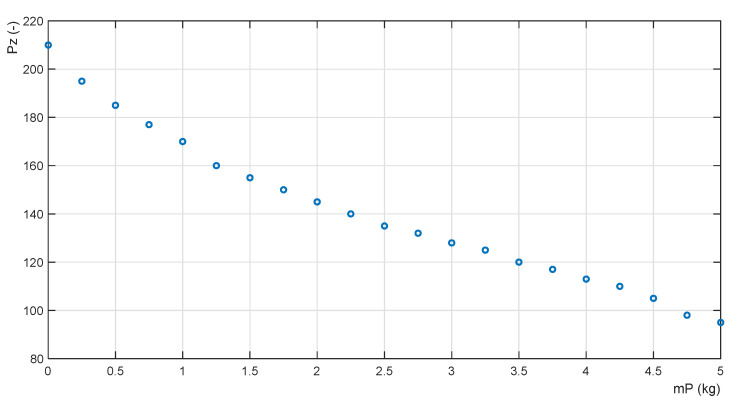
Adaptation function $P_{Z},opt=f\left(mP\right)$.

Function (20) represents an adaptation function that assigns the optimal value of the proportional gain of the controller $P_{Z},opt$ to each payload mass mP to achieve the minimum error $Err_{Z},min$ (Figure 9).

**Figure 10:**
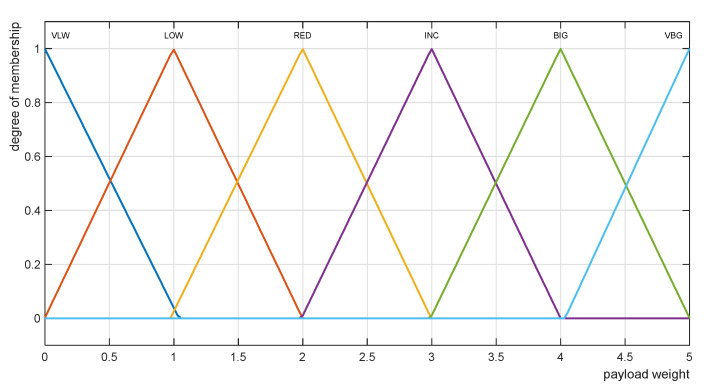
Membership function of linguistic variable mP.

To obtain an analytical expression of the nonlinear Function (20), the Takagi–Sugeno type approximation fuzzy model [28] was used. The structure of the model consists of a set of conditional IF-THEN rules; their parameters are identified by learning from the values of the training dataset (Table 2) by a multilayer neural network ANFIS [32]. The Takagi–Sugeno type fuzzy rule-based model comprises six conditional IF-THEN rules with six approximating linear functions in their consequences. The fuzzy interval of the size of the input variable in the antecedent of the rule defines the validity of a partial linear approximation function. The triangular approximations of the membership functions of the linguistic values of the input variable mP are shown in Figure 10.

The rules set of the nonlinear fuzzy TS model has the form:

(21) IF (mP is VLW), THEN PZ(1)=59.06+210.16mPIF (mP is LOW), THEN PZ(2)=82.66+86.55mPIF (mP is RED), THEN PZ(3)=90.50−34.73mPIF (mP is INC), THEN PZ(4)=98.30−165.46mPIF (mP is BIG), THEN PZ(5)=99.10−283.38mPIF (mP is VBG), THEN PZ(6)=102.10−415.88mP

The linguistic values of the input variable mP are: VLW—Very Low, LOW—Low, RED—Reduced, INC—Increased, BIG—Big, VBG—Very Big.

**Figure 11:**
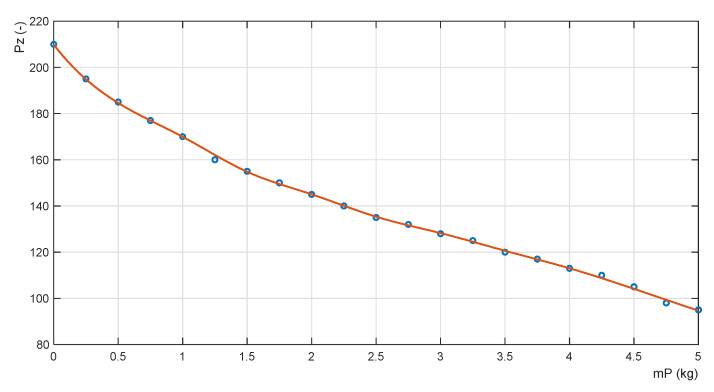
Approximated adaptation function.

The global value of the language model output variable $P_{Z,opt}$ is given by the relation of the weighted sum of the output values $P_{Z}\left(i\right)$ of each rule [28]

(22) $$P_{Z,opt}=\frac{\sum_{i=1}^{6}w\left(i\right).P_{Z}\left(i\right)}{\sum_{i=1}^{6}w\left(i\right)},$$

where the mass value w(i) is the truth value of the fuzzy logic statement in the antecedent of the i-th rule for the current value of the input variable mP. The approximated continuous nonlinear adaptation function has the form shown in Figure 11. The root mean square error of its approximation is RMSE = 0.337.

**Figure 12:**
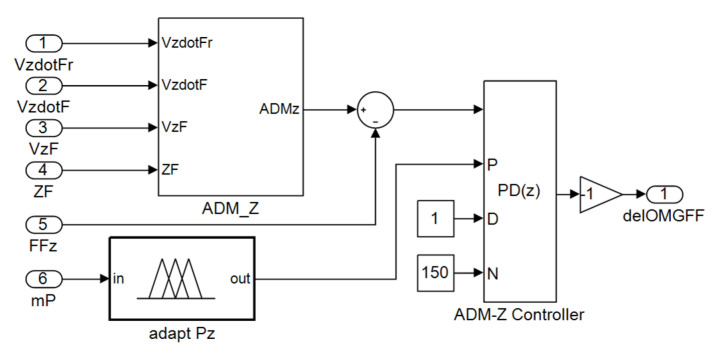
Admittance adaptive controller *ADM-Z*.

The adaptive admittance controller of the QDR-F ascent includes an adaptive fuzzy block adapt $P_{Z}$ (Figure 12), which calculates the optimal value of the proportional gain $P_{Z,}opt$ for the current mass of the transported payload mP using the fuzzy Model (21), (22).

The outputs of the admittance controllers are used as inputs to the controllers of the inner position control loop QDR-F (Figure 2).

## 6. Simulation Results

The function of the dynamic modelling and collaborative control system of tandem QDRs, implemented in the Matlab-Simulink programming environment [33], was tested by simulation calculations. The following values of their parameters were used for the dynamic models [18]: mQL = 1 kg, mQF = 1 kg, Kt = 0.14 × 10^−6^ N/rpm^−2^, Km = 4.6 × 10^−9^ Nm/rpm^−2^, l = 0.21 m, Ixx = 0.15 kg·m^2^, Iyy = 0.15 kg·m^2^, Izz = 0.22 kg·m^2^.

**Figure 13:**
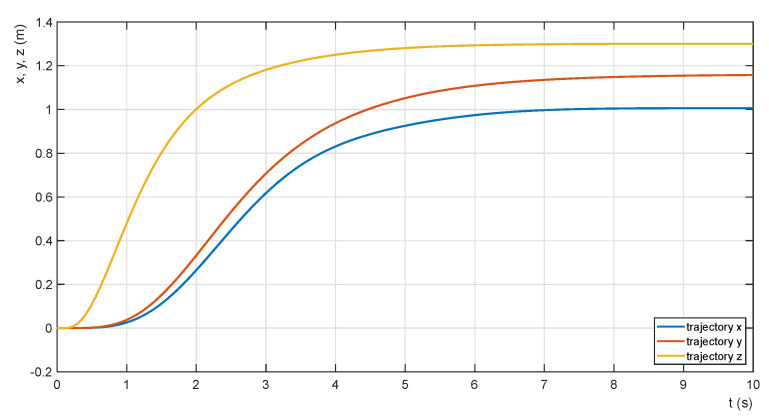
Motion trajectories on x-, y - and z -axes.

The collaborative admittance force feedback control system is designed to meet the conditions of transporting the payload in a horizontal position without abrupt changes in speed and direction of movement. Therefore, the time waveforms of the motion trajectories of the two QDRs and the synchronization of their motion on the x-, y and z=-axes were mainly investigated (Figure 13). All time series are obtained from a system with a payload mP = 5 kg and a z-axis proportional gain of the admittance controller value $P_{Z}$ = 200 (not corresponding to the optimal value). The parameters of the x- and y-axes position controllers are set to expert values for the simulation calculations so that large values or changes in the tilt values of the payload are excluded during transport. The required transfer values in each axis are set to *z* = 1.3 m, *x* = 1.15 m and *y* = 1.0 m. In order to achieve smooth motion, the primary step changes in the desired values are adjusted by the second-order transfer terms. The requirement to avoid abrupt changes in the speed of the tandem QDRs motion is further fulfilled by adjusting the parameters of the QDR-L motion PD controllers. The settling time of the motion control path on all axes is about t = 8 s. The value is practically satisfactory; its shortening is not a priority in terms of the requirement of smooth transport of the payload.

**Figure 14:**
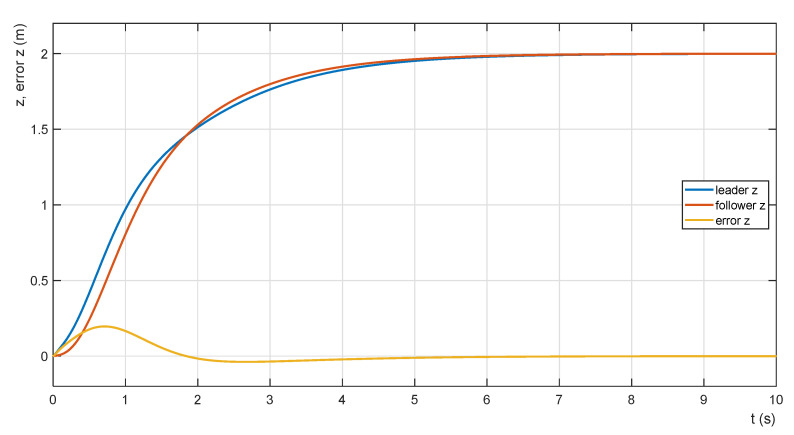
Ascent trajectories and error functions.

The difference (error) of the QDR-L and QDR-F position on the z-axis (18) represents the degree of stabilization of the payload in the horizontal direction, and it is the minimized value (adaptation of the transfer of the admittance controller). The relationship between the shape of the trajectories and the waveform of the horizontal position error of the payload during the ascent is shown in Figure 14. The courses are obtained for mP = 5 kg and $P_{Z}$ = 200.

**Figure 15:**
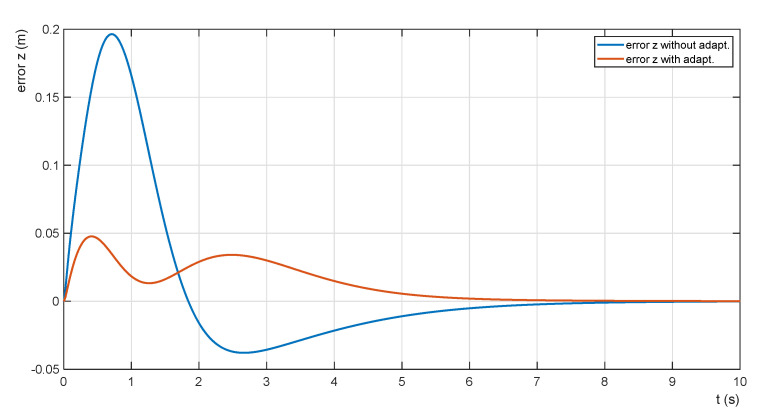
The effect of adaptation on the trajectories’ errors.

The minimization of the horizontal attitude error is performed by adapting the size of the proportional gain of the adaptive ascent controller depending on the actual mass of the transported payload. The relationship between the trajectory shape and the error is shown in Figure 15.

The functions are obtained for a payload mass of mP = 5.0 kg. The horizontal position error is reduced by adaptation from Err,z = 0.19 m to $Err_{Z},min$ = 0.04 m.

**Figure 16:**
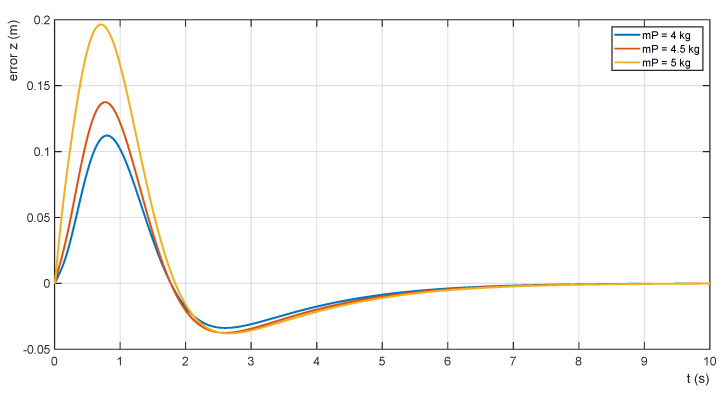
Error functions without adaptation of $P_{Z}$.

**Figure 17:**
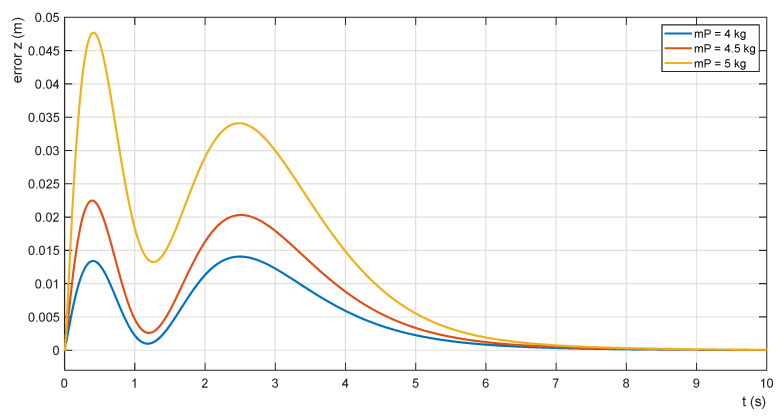
Error functions with adaptation of $P_{Z}$.

The effectiveness of the adaptation procedure is shown also in Figure 16 and Figure 17. The waveforms of the parametric error functions for mP = 5.00 kg, 4.50 kg and 4.00 kg without adaptation are shown in Figure 16. As the mass of the payload decreases, the horizontal position error decreases as well. Its minimum values are given in Table 3. The error function waveforms for the same payload masses with the adaptation of the admittance controller are shown in Figure 17.

The values of the errors are listed in Table 3. The examples given demonstrate a significant reduction in the error of the horizontal position of the payload, especially for higher values of its mass.

## 7. Discussion

Using the leader–follower control strategy, only the positional information of the leader QDR is needed. Motion tracking cameras are used in indoor environments, or the Global Position System (GPS) for outdoor environments is suitable. The GPS-RTK system, supplemented by a stationary station, provides position determination with an accuracy of ±1 cm.

When using the FFC approach, rigid grasping of the payload by both QDRs is used. Then, the follower QDR-F is led by the interaction contact force and torques acting on it, measured through the force/torque sensors located at the contact point of the follower QDR-F and the payload. The most important aspect is that the follower QDR-F can be controlled without the use of GPS or visual-inertial navigation control. In addition, no data communication is needed between QDRs.

We can use several modern miniature sensors to obtain information about the QDR movement parameters [34]. There are some modern technologies that support the production of individual or integrated suitable sensors systems. An IMU (Inertial Measurement Unit) is a specific type of sensor that measures the angular rate force. IMUs are composed of a three-axis accelerometer and a three-axis gyroscope [35]. Increased accuracy is provided by IMU sensors using the AHRS algorithm. For applications where stability is paramount, tilt sensors combined with gyros and accelerometers are suitable (MEMSIC). In drones, power consumption is also important. Current sensors can monitor and optimize power drain, safe charging of internal batteries, and detect fault conditions with motors or other areas of the system [36]. The monitoring of an important parameter—the vibration of their rotors—is an interesting aspect of the reliability of particularly large quadrotors. A diagnostic system using nonlinear regression models is shown in [37].

There may also be question about the possible influence of external forces and influences on the quadrotors. The “QDRs-rigid load” transport system is fixed. Therefore, the mutual position of both QDRs cannot be influenced by any forces. Even if the aerodynamic forces between QDR-L and QDR-F or other aerodynamic forces caused by other flying objects act, they would, like other external disturbances, be compensated by quadrotors’ controllers.

## 8. Conclusions

The paper presents the issue of modelling and controlling a system of two QDRs performing the task of transporting a common payload. The solution is based on the leader–follower approach using the admittance Force Feedback Control scheme method. The solution requires rigid grasping of the payload by both QDRs. The admittance follower controller simulates a virtual “mass-spring-damper” system and generates a force compliant desired trajectory for the follower. The force feedback control method eliminates the need for external position tracking of the QDRs and does not require data communication between them. This paper presents a dynamic QDR model for computing contact forces and torques. The priority control objective of the proposed collaborative system is to stabilize the horizontal position of the payload during its transport and the associated motion mode adjustment with the limitation of abrupt changes in speed and direction of motion. The minimization of the payload horizontal position error is achieved by using fuzzy approximations of the adaptive transfer function of the admittance controller of the follower vertical motion. The tilt of the QDRs during motion changes along the *x*- and *y-* axes, respectively, is small because the desired trajectories are tracked without abrupt changes in their speed and direction (Figure 13). The collaborative transport system was designed in the Matlab-Simulink environment. Simulation calculations confirmed the effectiveness of the system function.

## Figures and Tables

**Table 1:** Data of objective function (mP = 5 kg).

*P_Z_*	40	45	50	55	60	65	70	75	80	85	90	95
*Err,z* (cm)	22.4	19.4	16.2	13.8	11.8	10.0	8.5	7.1	6.0	5.0	4.2	4.8
*P_Z_*	100	105	110	115	120	125	130	135	140	145	150	155
*Err,z* (cm)	5.5	6.2	7.1	8.0	8.7	9.75	10.6	11.4	12.2	13.0	13.7	14.4
*P_Z_*	160	165	170	175	180	185	190	195	200	205	210	160
*Err,z* (cm)	15.1	15.7	16.3	16.9	17.5	18.0	18.6	19.1	19.8	20.4	21	15.1

**Table 2:** Data of adaptation function $P_{Z},opt=f\left(mP\right)$.

*mP* (kg)	0.00	0.25	0.50	0.75	1.00	1.25	1.50	1.75	2.00	2.25	2.50
$P_{Z},opt$	210	195	185	177	170	160	155	150	145	140	135
*mP* (kg)	2.75	3.00	3.25	3.50	3.75	4.00	4.25	4.50	4.75	5.00	
$P_{Z},opt$	132	128	125	120	117	113	110	105	98	95	

**Table 3:** Horizontal position error minimization.

mP (kg)	$P_{Z}$	Err,z (cm)	$P_{Z},opt$	Err,z,min (cm)
5.00	200	19.5	95	4.2
4.50	200	13.7	98	2.2
4.00	200	11.2	113	1.4
